# The ABC of Ribosome-Related Antibiotic Resistance

**DOI:** 10.1128/mBio.00598-16

**Published:** 2016-05-03

**Authors:** Daniel N. Wilson

**Affiliations:** a Gene Center and Department of Biochemistry, University of Munich, Munich, Germany; b Center for Integrated Protein Science Munich (CiPSM), Munich, Germany

## Abstract

The increase in multidrug-resistant pathogenic bacteria is limiting the utility of our current arsenal of antimicrobial agents. Mechanistically understanding how bacteria obtain antibiotic resistance is a critical first step to the development of improved inhibitors. One common mechanism for bacteria to obtain antibiotic resistance is by employing ATP-binding cassette (ABC) transporters to actively pump the drug from the cell. The ABC-F family includes proteins conferring resistance to a variety of clinically important ribosome-targeting antibiotics; however, controversy remains as to whether resistance is conferred via efflux like other ABC transporters or whether another mechanism, such as ribosome protection, is at play. A recent study by Sharkey and coworkers (L. K. Sharkey, T. A. Edwards, and A. J. O’Neill, mBio 7:e01975-15, 2016, http://dx.doi.org/10.1128/mBio.01975-15) provides strong evidence that ABC-F proteins conferring antibiotic resistance utilize ribosome protection mechanisms, namely, by interacting with the ribosome and displacing the drug from its binding site, thus revealing a novel role for ABC-F proteins in antibiotic resistance.

## COMMENTARY

Antibiotics prevent bacterial growth by targeting fundamental processes of the cell, ranging from cell membrane biogenesis, to DNA and RNA replication, to protein synthesis (1). Indeed, the ribosome and protein synthesis represent one of the major targets for clinically used antibiotics, for example macrolides, streptogramins, lincosamides, pleuromutilins, oxazolidinones, and phenicols (2, 3). Bacteria utilize a wide range of different mechanisms to obtain resistance to this arsenal of ribosome-targeting antibiotics (3). Target alteration, such as mutation or modification of the drug-binding site on the ribosome, is employed by many bacteria to obtain antibiotic resistance; however, this mechanism often comes with a high fitness cost due to reduced functionality of the highly conserved centers of the ribosome. Alternatively, bacteria can obtain resistance via efflux or ribosome protection mechanisms, which do not require manipulation of the conserved translational machinery.

Active efflux of antibiotics by bacteria utilizes a wide range of membrane transporters where drug efflux is either coupled to an electrochemical gradient, or, alternatively, ATP hydrolysis is used an energy source. The latter ATP-binding cassette (ABC) transporters consist of two cytosolic nucleotide-binding domains (NBDs), which bind and hydrolyze ATP, linked to transmembrane domains (TMDs) that anchor the protein in the membrane and facilitate drug expulsion. Based on the sequence similarity of the NBDs, the ABC protein family has been divided into eight subfamilies, denoted by the letters A to H (4). Unlike the other subfamilies, the ABC-E and ABC-F subfamilies lack any identifiable TMDs, suggesting that these proteins may not be directly involved in transport (5, 6). Indeed, many members of this class have well-characterized roles in other intracellular processes, including DNA repair and replication and translation regulation (7). The recent study by Sharkey and coworkers further extends the non-transport-related function of the ABC-F subfamily by demonstrating these proteins can confer resistance to many ribosome-targeting antibiotics through interaction with the ribosome and displacement of the drug from its binding site (8).

The antibiotic resistance (ARE) ABC-F proteins are found in Gram-positive antibiotic-producing bacteria, such as *Streptomyces*, as well as pathogenic bacteria, such as *Staphylococcus*, *Streptococcus*, and *Enterococcus* (5). ARE ABC-F proteins have been identified that confer resistance to antibiotics that bind at or near the peptidyl-transferase center (PTC) of the ribosome. Three main distinctions have been made based on the available resistance profiles, namely, the (i) Vga/Lsa/Sal type conferring resistance to lincosamides, streptogramins A, and sometimes pleuromutilins, (ii) the Msr type conferring resistance to macrolides and streptogramins B, and (iii) the OptrA type conferring resistance to phenicols and oxazolidinones (8).

The study by Sharkey and coworkers (8) addresses the mechanism of action of the Vga/Lsa/Sal-type ABC-F proteins, providing the first direct evidence that these ABC-F proteins confer resistance by acting directly on the ribosome, as suggested previously (5, 6, 9). Specifically, the authors demonstrated that the addition of purified *Staphylococcus aureus* Vga(A) protein to an *S. aureus* in vitro coupled transcription-translation system relieved the inhibition caused by the streptogramin B antibiotic virginiamycin M (8). Interestingly, while Lsa(A) from *Enterococcus faecalis* also displayed rescue activity against virginiamycin M and lincomycin in the *S. aureus* translation system, Vga(A) and Lsa(A) did not appear to be active on *Escherichia coli* ribosomes, suggesting some species-specific interactions are required for ribosome binding (8).

Moreover, the presence of Lsa(A) reduced the binding of radiolabeled lincomycin to *S. aureus* ribosomes (8), indicating that the ABC-F proteins induce dissociation of the drug from the ribosome. The reactions were performed with ATP, and the effect of nonhydrolyzable analogs, such as ADPNP (5′-adenylyl-β,γ-imidodiphosphate), was not analyzed; therefore, it remains to be determined as to whether drug release requires ATP hydrolysis or whether ATP hydrolysis is only required for subsequent dissociation of the ABC-F protein from the ribosome. What is clear is that the ATPase activity of Vga(A) is critical for resistance since mutation of the catalytic glutamines within the NBDs of Vga(A) did not confer resistance *in vivo* (10) nor rescue activity in the *in vitro* translation system (8).

One of the major questions remaining open relates to the mechanism by which the ABC-F proteins promote drug dissociation from the ribosome, namely, as to whether the ABC-F proteins physically overlap the drug-binding sites on the ribosome or whether binding of the ABC-F proteins to the ribosome induces conformational changes in the ribosome that promote drug release allosterically. In this regard, it is interesting to note that mutations within the linker region connecting the two NBDs can alter the efficiency, as well as specificity, of antibiotic resistance (8, 9, 11). For example, wild-type Vga(A) confers low-level resistance to lincosamides, whereas a K219T mutation within the linker region increases the effectiveness against lincomycin *in vivo* (9) and *in vitro* (8).

**FIG 1:**
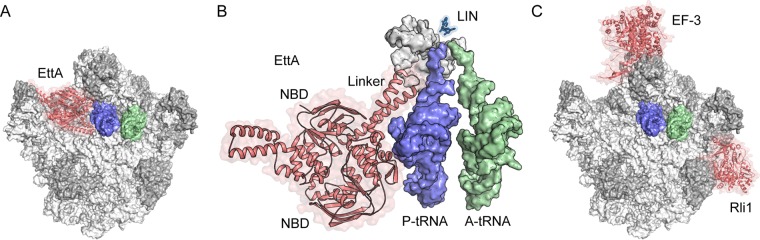
Interaction of ABC proteins with the ribosome. (A) Overview of the binding site of EttA (red) on the ribosome (white, RNA; gray, proteins) (12) relative to P-tRNA (blue) and A-tRNA (green). The small ribosomal subunit is omitted for clarity. (B) The linker region of EttA approaches but cannot access the PTC of the ribosome, where the CCA end of P-tRNA (blue), A-tRNA, and antibiotics, such as lincomycin (LIN [blue]) (17), interact. (C) Relative binding positions of EF-3 (13) and Rli1 (14) on the ribosome relative to A- and P-tRNAs.

Based on a cryo-electron microscopy (cryo-EM) structure of the ABC protein EttA in complex with the ribosome (12), as well as the high similarity between Vga(A) and EttA, a model for Vga(A) on the ribosome was proposed (9) (Fig. 1A). This model suggests that the linker region of Vga(A), which is extended by 30 amino acids (aa) compared to EttA, would approach the drug-binding site at the PTC of the ribosome (9); however, direct access of ABC-F to the drug would be precluded by the presence of a P-site tRNA (Fig. 1B). Since the antibiotics to which the ARE ABC-F proteins confer resistance, such as lincomycin (and chloramphenicol), are elongation inhibitors and trap the ribosome with a P-tRNA in the P-site (2), it is hard to envisage based on this model a direct mechanism of drug removal by the ABC-F proteins. Alternatively, the ARE ABC-F proteins may utilize a different mode of interaction with the ribosome, such as seen for the eukaryotic ABC proteins EF-3 (13) and Rli1 (14) (Fig. 1C). A novel mode of binding of the ARE ABC-F proteins, such as VgaA and Lsa(A), in the A-site of the ribosome would enable the linker region to directly access the PTC and tunnel region of the ribosome, even in the presence of a P-tRNA. Structures of ARE ABC-F factors in complex with the ribosome will be needed to address these points and provide insight into mechanism of action of how these proteins dislodge the antibiotic from the ribosome.

In conclusion, it appears that the ARE ABC-F proteins may work analogously to the TetM/TetO ribosome protection proteins, which confer resistance to tetracyclines by binding and chasing the drug from its binding site on the ribosome (15, 16). As with TetM/TetO, a number of important questions still remain unanswered: For example, it remains unclear as to what prevents the drugs from rebinding following or during dissociation of the ABC-F proteins from the ribosome. Also drug rebinding would imply that multiple cycles of ARE ABC-F-mediated drug release would be required to fully translate a protein, which has yet to be shown. Understanding the mechanism by which bacteria obtain resistance to antibiotics will help the future development of improved antimicrobial agents that overcome multidrug-resistant bacteria.
